# Comments on the Impact of COVID-19 Pandemic on Hepatocellular Carcinoma Surveillance in the United States

**DOI:** 10.14309/ctg.0000000000000767

**Published:** 2024-10-29

**Authors:** Salvatore Chirumbolo, Umberto Tirelli

**Affiliations:** 1 Department of Engineering for Innovation Medicine, University of Verona, Verona, Italy;; 2 Tirelli Medical Group, Pordenone and Former Head of the Oncology Unit of the National Cancer Center, Aviano (PN), Italy.

The recent contribution by Liang et al in this journal (1), as recently commented on by Mattiuzzi and Lippi (2), investigated the rate of underdiagnosis of hepatocellular carcinomas (HCCs) due to reduced cancer surveillance in the United States during the coronavirus disease 2019 pandemic (1). The authors reported that, grounding on their population-based survey, the incidence rates of HCC in the United States were significantly lower in 2020 compared with prepandemic rates (1), likely due to the decreased diagnostic activity during that time. However, Mattiuzzi and Lippi noted that no significant difference could be observed when using data from the US Centers for Disease Control and Prevention Wide-ranging Online Data for Epidemiologic Research database for the period 2018–2022.

It is possible that the statistical analysis of Mattiuzzi and Lippi may have introduced some bias.

However, the data reported by Liang et al were sourced from the US Cancer Statistics database, which includes the National Program of Cancer Registries and Surveillance, Epidemiology, and End Results databases. Therefore, it seems odd to critique their findings using a different database (1,2).

Mattiuzzi and Lippi reported using analysis of variance (ANOVA) with a Tukey post hoc test but did not sufficiently reassure readers about the validity of their approach. Before conducting a Tukey test, it is essential to confirm that the assumptions of ANOVA—normality, equal variances, and independence—are met. The authors did not assess normality, which is a crucial step since both ANOVA and Tukey test assume normality and equal variances. If these assumptions are violated, the results may be unreliable (1,2).

When analyzing the number of HCC cases in the US population from 2018 to 2022, it is more likely that the data follow a nonparametric distribution rather than a normal distribution. The number of HCC cases per year is count data, which typically follows a Poisson distribution or a negative binomial distribution, especially in cases of overdispersion (where variance exceeds the mean). Count data rarely adhere to a normal distribution because it is discrete and nonnegative. Health-related data, such as cancer incidence, often show right skewness due to factors such as outbreaks, environmental changes, or improved detection methods. Variations in public health interventions, screening programs, or reporting practices can create outliers, making the data deviate from normality. In addition, the number of HCC cases can vary significantly due to changes in risk factors (e.g., hepatitis C infection rates, alcohol use, and obesity), advancements in diagnostic techniques, or shifts in population demographics. Such variability makes a normal distribution less likely. Given this, nonparametric methods or other suitable models (such as Wilcoxon, Poisson, or negative binomial regression) may be better suited for analysis, as they do not assume normality and can handle skewed data or outliers more effectively.

In conclusion, the data reported by Liang et al may indeed be accurate and consistent with other findings reported in the literature (3).

## CONFLICTS OF INTEREST

**Guarantor of the article:** Salvatore Chirumbolo, PhD.

**Specific author contributions: **S.C.: conceived the manuscript, managed and performed the research. retrieved bibliography, wrote and validated the manuscript, submitted the manuscript; U.T.: supervised the manuscript, revised the manuscript, validated the manuscript.

**Financial support:** None to report.

**Potential competing interests:** None to report.
